# Examination of wnt signaling mediated melanin transport and shell color formation in Pacific oyster (*Crassostrea gigas*)

**DOI:** 10.1007/s42995-024-00221-5

**Published:** 2024-06-06

**Authors:** Yue Min, Qi Li, Hong Yu, Shaojun Du

**Affiliations:** 1 Key Laboratory of Mariculture, Ministry of Education, and College of Fisheries, Ocean University of China, Qingdao, 266003 China; 2 Laboratory for Marine Fisheries Science and Food Production Processes, Qingdao National Laboratory for Marine Science and Technology, Qingdao, 266237 China; 3 Institute of Marine and Environmental Technology, Department of Biochemistry and Molecular Biology, University of Maryland School of Medicine, Baltimore, 21240 USA

**Keywords:** Melanogenesis, Melanosome trafficking, WIF-1

## Abstract

Mollusca exhibit remarkable diversity in shell coloration, attributed to the presence of melanin, a widely distributed pigment with various essential roles, such as mechanical strengthening, antioxidation and thermoregulation. However, the regulatory network governing melanogenesis and melanin transport in molluscs remains poorly understood. In this study, we conducted a systematic analysis of melanin distribution and transport in the Pacific oyster, utilizing light microscopy and high-resolution transmission electron microscopy. In addition, we characterized *CgWnt1* and *CgWnt2b-a* in *Crassostrea gigas,* and analyzed Wnt signaling in melanocyte formation. Expression analysis revealed that these genes were predominantly expressed in the mantle of black-shelled individuals, particularly in the outer fold of the mantle. Furthermore, we employed RNA interference and inhibitors to specifically inhibit Wnt signaling in both in vivo and in vitro. The results revealed impaired melanogenesis and diminished tyrosinase activity upon Wnt signaling inhibition. These findings suggest the crucial role of Wnt ligands and downstream factors in melanogenesis. In summary, our study provides valuable insights into the regulatory mechanism of shell pigmentation in *C. gigas*. By demonstrating the promotion of melanogenesis through Wnt signaling modulation, we contribute to a better understanding of the complex processes underlying molluscan melanin production and shell coloration.

**Supplementary Information:**

The online version contains supplementary material available at 10.1007/s42995-024-00221-5.

## Introduction

The black coloration of feathers, fur, shells and skin is largely determined by melanocytes. Alongside tetrapyrroles and carotenoids, melanin plays a significant role in pigmentation. Melanin exists as polymers composed of two monomer molecules, indolequinone and dihydroxyindole (Bandaranayake 2006), which are produced in melanocytes through a process called melanogenesis (Tapia et al. 2014). These melanin-containing granules, known as melanosomes, are unique lysosome-related organelles that synthesize two types of melanin: reddish-yellow pheomelanin and brownish-black eumelanin (Thody et al. 1991). Melanin synthesis begins with the substrate tyrosine, which is converted into L-DOPA, and then oxidized to dopaquinone, both reactions catalyzed by the pigment cell-specific enzyme tyrosinase (Hearing and Jiménez 1987). After melanin synthesis, mature melanosomes are transported to neighboring keratinocytes (Costin and Hearing 2007). Melanogenesis is triggered and regulated by numerous signaling molecules and transcription factors.

Microphthalmia-associated transcription factor (MITF) is the primary regulator of melanogenesis. It plays a crucial role in the survival and proliferation of melanoblasts derived from the neural crest, as well as in the regulation of melanocyte differentiation by stimulating key melanogenesis enzymes, including tyrosinase (Tyr), tyrosinase-like protein 1 (Tyrp1) and dopachrome tautomerase (DCT) (Carreira et al. 2006; Hornyak et al. 2001; Steingrímsson et al. 2004). Multiple signaling pathways can regulate MITF, such as the Wnt/β-catenin pathway (Gajos-Michniewicz and Czyz 2020), phosphatidylinositol 3-kinase/protein kinase B (PI3K/AKT) pathway (Su et al. 2013), mitogen-activated protein kinases/extracellular signal-regulated kinase (MAPK/ERK) pathway (D'Mello et al. 2016) and the cyclic adenosine monophosphate (cAMP)-mediated pathway (Buscà and Ballotti 2000). The Wnt/β-catenin pathway is particularly important in melanocyte development (Zhang et al. 2013). This pathway is initiated by the binding of Wnt ligands to their receptors, Frizzled, and co-receptors low-density lipoprotein-receptor-related protein 5 and 6 (LRP5/6) (MacDonald and He 2012). Activation of the Wnt ligand-receptor complex leads to the inhibition of glycogen synthase 3β (GSK-3β) phosphorylation, resulting in the accumulation of cytoplasmic β-catenin (Liu et al. 2022). The accumulated β-catenin is then stabilized and transported into the nucleus, where it binds to the lymphoid-enhancing factor (LEF/TCF) transcription factor, thereby increasing the expression of MITF (Kim et al. 2013b; Nusse and Varmus 2012; Rim et al. 2022; Wu et al. 2003).

Studies in mice deficient in both Wnt1 and Wnt3α have demonstrated a deficiency in DCT, a crucial enzyme in melanogenesis (Dunn et al. 2000). Further research has confirmed that Wnt1 instructs melanoblasts to increase the number of melanocytes, while Wnt3α and β-catenin maintain the expression of MITF and promote the development of neural crest cells into melanocytes (Dunn et al. 2005; Jin et al. 2000). In zebrafish, exposure to LiCI (Wnt enhancer) and W-C59 (Wnt inhibitor) was observed to induce either a stimulatory or defective effect on melanocyte development (Silva and Atukorallaya 2023). β-catenin is the central effector in the Wnt signaling, functioning as a transcription activator (Rim et al. 2022). In melanocytes, β-catenin can interact with LEF1 to synergistically regulate the MITF expression (Takeda et al. 2000). β-catenin’s involvement in melanoblast determination has been directly observed in various species through gain- and loss-of-function studies. For example, in hair follicles of animals, the gain-of-function mutation of β-catenin in the dermal papilla can lead to a black phenotype (Enshell-Seijffers et al. 2010). In mice, Cre-mediated knock-out of β-catenin in adult melanocyte stem cells (McSCs) leads to permanent whitening of the fur or hair (Le Coz et al. 2021). By activating β-catenin expression, the facilitation of schwann cell precursors commitment towards the melanocyte lineage ensues (Colombo et al. 2022).

Mollusca is a highly diverse group of animals renowned for their vibrant and remarkable shells. The establishment of melanogenesis regulation networks in marine invertebrates remains poorly understood. The Pacific oyster *Crassostrea gigas*, known for its high economic value, is among the most widely cultured shellfish, worldwide (Teixeira Alves et al. 2021). The shell color, serving as a distinctive trait, plays a significant role in enhancing the commercial value of oysters. However, the mechanisms underlying pigmentation in *C. gigas*, from molecular processes to the overall system, remain elusive. Consequently, it is crucial to uncover the essential molecular pathways involved in melanin synthesis.

This study aimed to explore the potential involvement of the Wnt/β-catenin pathway in melanogenesis regulation in *C. gigas*. To investigate this, we employed methods, such as the inhibition of the Wnt/β-catenin pathway and knockdown of key genes. The results provide valuable insights into the molecular mechanisms underlying melanogenesis regulation in *C. gigas*, particularly from the perspective of Wnt signal transduction.

## Materials and methods

### Animals and sampling

Black and white shell oysters were obtained from an oyster farm in Weihai, Shandong, China, and were maintained in seawater at 20 °C for one week prior to experimentation. Six tissues, including the gill, digestive gland, adductor, labial palp, edge mantle, and central mantle, were promptly dissected, flash-frozen in liquid nitrogen, and stored at -80 °C for RNA extraction. *C. gigas* embryos and larvae from the black shell color strain were obtained from our previous study (Min et al. 2022a, b). For in situ hybridization, edge mantles were taken and fixed in 4% paraformaldehyde in phosphate-buffered saline (PBS) for 12 h at 4 °C. The samples were then dehydrated and preserved in methanol at − 20 °C until further use.

### RNA extraction and Relative mRNA quantification

Total RNA was extracted using TRlzol (Invitrogen, USA) according to the manufacturer's instructions. The quality, concentration, and integrity of the RNA were determined using the Nanodrop 2000 (Thermo, USA) and 1.5% agarose gel electrophoresis. The reverse transcription of total RNA (1 µg) was performed using the HiScript III 1st strand cDNA synthesis kit (Vazyme, China).

To assess the expression profiles of *CgWnt1* and C*gWnt2b-a*, qPCR primers were designed using Primer Premier 5.0 software (Supplementary Table S1). The QuantiNova SYBR Green PCR kit (Qiagen, Germany) was used for qPCR, which was performed on a Lightcycler 480 real-time PCR instrument (Roche, Switzerland). The reaction was conducted in a 10 µL volume, including 5 µL of 2× SYBR Green Master Mix, 1 µL of cDNA template, 0.2 µL each of 10 µmol/L primers, and 3.6 µL of RNase-free water. In adult samples, elongation factor 1-α (ef1α) was used as an internal control (Li et al. 2021). Elongation factor 1-α (ef1α), adp-ribosylation factor 1 (arf1), and glyceraldehyde-3-phosphate dehydrogenase (gaph) were used as internal controls in larval samples (Huan et al. 2016). The 2^- ΔΔCT^ method was used to calculate relative expression.

### Phylogenetic analysis of CgWnt1 and CgWnt2b-a

The amino acid sequences of Wnt1 and Wnt2b-a were obtained from the NCBI database. A phylogenetic tree was constructed using the neighbor-joining (NJ) method with the Jones Taylor-Thornton (JTT) + Gamma Distributed (G) model in MEGA 7. Motif analysis was conducted using the MEME Suite (https://meme-suite.org/meme/). The GeneBank accession numbers for the phylogenetic analysis are listed in Supplementary Table S2.

### Histological observation

Mantle tissues were fixed in Bouin’s fluid (Sbjbio Life Science, Nanjing) for 24 h, decolorized with 70% ethanol, and then stored in 70% ethanol at room temperature. Subsequently, the tissues were dehydrated using a gradient of ethanol, embedded in paraffin, sectioned at 5 µm, and stained with hematoxylin dye for histological observation using an Olympus BX53 light microscope (Olympus Corporation, Japan).

Melanin staining was performed using a ferrous sulfate staining kit (Leagene, DJ002, China) following the protocols provided by the manufacturer with slight modifications. Briefly, the dewaxed and rehydrated paraffin-embedded sections were sequentially immersed in xylene and gradient ethanol (95%, 80%, 70%, 50%) for 4 min each time. Subsequently, the sections were stained with ferrous sulfate solution and acid potassium ferricyanide solution for 45 min each. After each staining solution, the sections were rinsed three times with ultrapure water for 4 min. The sections were then counterstained with nuclear fast red solution for 10 min and photographed with an Olympus BX53 light microscope after being washed.

### In situ hybridization

The primers designed for the sense and antisense probes are listed in Supplementary Table S1. Probe synthesis was performed using a DIG RNA labeling kit (Roche, Switzerland). For sense probe synthesis, a T7 primer sequence (GATCACTAATACGACTCACTATAGGG) was added in front of the forward primer. Conversely, the reverse primers also include the T7 primer sequence for antisense probe synthesis. After probe preparation, ISH was carried out on 5 μm thick sections. Following deparaffinization, prehybridization, hybridization, and antibody incubation, the sections were treated with a 2% NBT/BCIP solution (Roche, Switzerland) for color reaction at room temperature. The duration of the color reaction depended on the actual conditions. After washing with PBS, the color reactions were terminated, followed by counterstaining with 0.5% eosin, and examination using an Olympus BX53 microscope (Olympus Corporation, Japan) for ISH images.

### RNAi experiment

A total of sixty one-year-old black shell *C. gigas* oysters (shell length: 39.98 ± 6.08 mm; shell height: 54.92 ± 7.63 mm) were temporarily cultivated in tanks of seawater for the experiment. The oysters were randomly divided into three groups: the PBS group, Wnt1 double-stranded RNA (dsRNA) interference group, and Wnt2b-a dsRNA interference group.

Using the Wnt1 (GenBank accession LOC105348242) and Wnt2b-a (GenBank accession LOC105340175) cDNA sequences, two coding sequence fragments were selected to design the target sites for dsRNA in RNAi (Supplementary Table S1). A 585 bp fragment of Wnt1 and a 495 bp fragment of Wnt2b-a were amplified. The purified PCR-derived templates were used for in vitro transcription to produce dsRNA using the T7 RNAi Transcription Kit (Vazyme, China). The dsRNA was diluted to a concentration of 1 µg/µL using 0.1 mol/L PBS. For the RNAi experiment, each oyster in the interference groups was injected with 45 µL of dsRNA (45 µg) into the adductor muscle. In the blank control group, equal injections of PBS were administered, following the methodology of previous studies (Lv et al. 2021; Tian et al. 2021a). In the pre-experiment, twelve individuals from each group were injected, and mantles were collected at various time intervals (12 h, 24 h, 36 h, and 48 h) post-injection to determine the optimal interference time for the subsequent experiment. A total of three injections were performed on days 1, 3, and 5. Furthermore, mantle samples were frozen in liquid nitrogen on day 7 for mRNA quantification and tyrosinase activity analysis. Fresh mantle samples (1 mm^3^ volume) were fixed in 2.5% glutaraldehyde prior to transmission electron microscopy (TEM) analysis.”

### Plasmids construction, cell culture, transfection, and luciferase assays

The coding sequences of CgWnt1, CgWnt2b-a, CgWIF-1, Cgβ-catenin-like, and Cgβ-catenin-like protein 1 were subcloned into the pcDNA3.1( +) plasmid using the ClonExpress II One-Step Cloning Kit (Vazyme, Nanjing, China). 293 T cells were cultured in DMEM medium (Hyclone, USA) supplemented with 10% fetal bovine serum (Hyclone, USA) and 1% Penicillin–Streptomycin solution (Hyclone, USA) at 37 °C with 5% CO_2_. The 293 T cells were seeded in 24-well plates and grown to 70–80% confluence before transfection. For transfection, TOPFlash plasmid (500 ng) (Beyotime, China) was transfected into the cells using lipofectamine 3000 (Invitrogen, USA). CgWnt1 (250 ng) and CgWnt2b-a (250 ng) were co-transfected with Cgβ-catenin-like or Cgβ-catenin-like protein 1 plasmid (250 ng) to investigate the effect of β-catenin/TCF signaling. The pRL-TK plasmid (10 ng) (Promega, USA) was co-transfected as an internal control. To study the function of CgWIF-1, the CgWIF-1 plasmid (250 ng) was co-transfected with either CgWnt1 plasmid (250 ng) or CgWnt2b-a plasmid (250 ng). In each transfection, TOPFlash plasmid (500 ng) and pRL-TK plasmid (10 ng) were essential components. The FOPflash plasmid with mutant binding sites served as the negative control. After 48 h of transfection, luciferase activity was measured using the Dual-Luciferase Reporter Assay System (Promega, USA) according to the manufacturer’s protocol. The relative luciferase activity (TOPFlash activity: Renilla luciferase activity) was measured using the Synergy^™^ H1 microplate reader (BioTek, USA).

### In vitro culture of tissues and treatment with salinomycin and recombinant protein

In the in vitro experiment, one-year-old black shell oysters were immersed in sterilized seawater with 1× penicillin–streptomycin-gentamicin solution for 2 h before dissection. The edge mantle was dissected and rinsed with PBS (pH 7.4) three times, supplemented with 1× penicillin–streptomycin-gentamicin solution. The tissues were then rinsed with the primary medium, which consisted of a 1:1 mixture of L15 medium and M199 medium, supplemented with 10% fetal bovine serum and 1% 1× penicillin–streptomycin-gentamicin solution. The tissues were transferred into 1.5 mL centrifuge tubes and cut into pieces using sterilized scissors. Finally, the pieces were cultured in 12-well plates at 16 °C.

For treatment with salinomycin (Niwa et al. 2022), the product was added to the primary medium at final concentrations of 0, 10, 20, 30, and 40 µmol/L and maintained for 12 h. For treatment with recombinant protein WIF-1, the partial coding sequence of CgWIF-1 was subcloned into the pET32a plasmid. After sequencing, the WIF-1-pET32a plasmid was transformed into BL21 cells. Recombinant WIF-1 expression was induced with isopropylβ-D-thiogalactoside (IPTG) at a final concentration of 1 mmol/L to optimize the induced temperature and time (Supplementary Fig. S2). The purified protein was added to the primary medium at final concentrations of 0, 5, 10, 15, 20, and 50 µg/mL and maintained for 12 h. Afterwards, the medium was discarded, and the rinsed cells were collected for RNA or protein extraction, tyrosinase activity detection, and melanin detection.

### Subcellular localization of CgWnt1, CgWnt2b-a, and CgWIF-1

The coding sequences of CgWnt1, CgWnt2b-a, and CgWIF-1 were subcloned into pEGFP-N1 vectors (www.miaolingbio.com). HEK-293 T cells were seeded into confocal dishes (Leica, USA) and transfected with the recombinant expression vectors CgWnt1-pEGFP-N1, CgWnt2b-a-pEGFP-N1, or CgWIF-1-pEGFP-N1. After 48 h, the cells were stained with Dil (Beyotime, China) for 20 min in the dark. The images were captured and analyzed using an ultra-high-resolution laser confocal microscope, Leica TCS SP8 STED 3X, equipped with Leica Application Suite X software. The primers used for fusion vector construction are listed in Supplementary Table S1.

### Western blot analysis

Mantle tissues were homogenized with RIPA lysis buffer (Beyotime, China). After centrifugation, the supernatant was collected and quantified using a BCA protein assay kit (Beyotime, China). The protein samples were diluted with SDS-PAGE loading buffer (Solarbio, China) to adjust to the same concentration and then denatured at 95 °C for 10 min. The protein samples were separated by 12% SDS-PAGE and transferred to PVDF membranes (Beyotime, China). The membranes were blocked with 5% skimmed milk dissolved in TBST buffer for 2 h at room temperature. Subsequently, the membranes were incubated with diluted primary antibodies against CgTYR (1:1000) (Li et al. 2023), CgTYRP2 (1:1000) (Li et al. 2023), MITF (1:1500, ABclonal, China), β-catenin (1:1000, Fintest, China), p-β-catenin (1:1000, Fintest, China), and β-actin (1:1000, Beyotime, China) at 4 °C overnight. After washing with TBST buffer three times, the membranes were incubated with HRP-conjugated goat anti-rabbit IgG (1:1000, Beyotime, China) for 2 h at room temperature. The proteins were detected using enhanced chemiluminescence detection reagents (Vazyme, China) and captured using the GE ImageQuant LAS4000mini system (GE, USA).

### Tyrosinase activity assays

The tyrosinase activity of mantles was measured using the Tyrosinase Activity Assay Kit (Solarbio, China). For the tissue samples, 0.1 g of mantle tissue was homogenized with lysis buffer under ice water conditions. The supernatant was collected after centrifugation, and then mixed with the detection buffer at 25 °C for 50 min. The tyrosinase activity was determined using a spectrophotometer at 475 nm. The sample treatment conditions were different for cells. The cells were treated with lysis buffer in proportion to the number of cells. The remaining procedures were the same.

### Enzyme-linked immunosorbent assays

The melanin content was measured using an enzyme-linked immunosorbent assay (ELISA) kit from Ruixin, China, following the instructions provided by the manufacturer. For tissue samples, 0.1 g of mantle tissues were homogenized with PBS supplemented with a protease inhibitor under ice water conditions. After centrifugation, the supernatant was collected and used for further analysis. The supernatant was incubated with biotin labeling antigen on an ELISA plate at 37 ℃ for 1 h. Subsequently, the ELISA plate was incubated with an HRP-conjugated reagent at 37 ℃ for 30 min. After a chromogenic reaction in darkness for 15 min, the absorbance was measured at 450 nm using a Multimode Microplate Reader (Synergy H1, BioTek, USA).

### Transmission electron microscopy of mantles

The isolated mantle tissues, measuring 1 mm^3^, were fixed in 2.5% glutaraldehyde at 4 °C. Subsequently, the specimens were postfixed with osmic acid and dehydrated using a series of acetone solutions. Afterward, the specimens were embedded in EPON 812 resin at room temperature. Finally, ultra-thin sections with a thickness of 60 nm were stained with uranyl acetate and lead citrate, and images were captured using a JEM-1200EX transmission electron microscope (JEOL, Japan) operating at 80.0 kV.

### Statistical analysis

Statistical analysis was performed using GraphPad Prism 8.0 software, utilizing a one-way ANOVA test. The results were presented as means ± standard deviation (SD). Statistical significance was considered at a P-value of less than 0.05 (*P* < 0.05).

## Results

### Histological observation of mantle

**Fig. 1:**
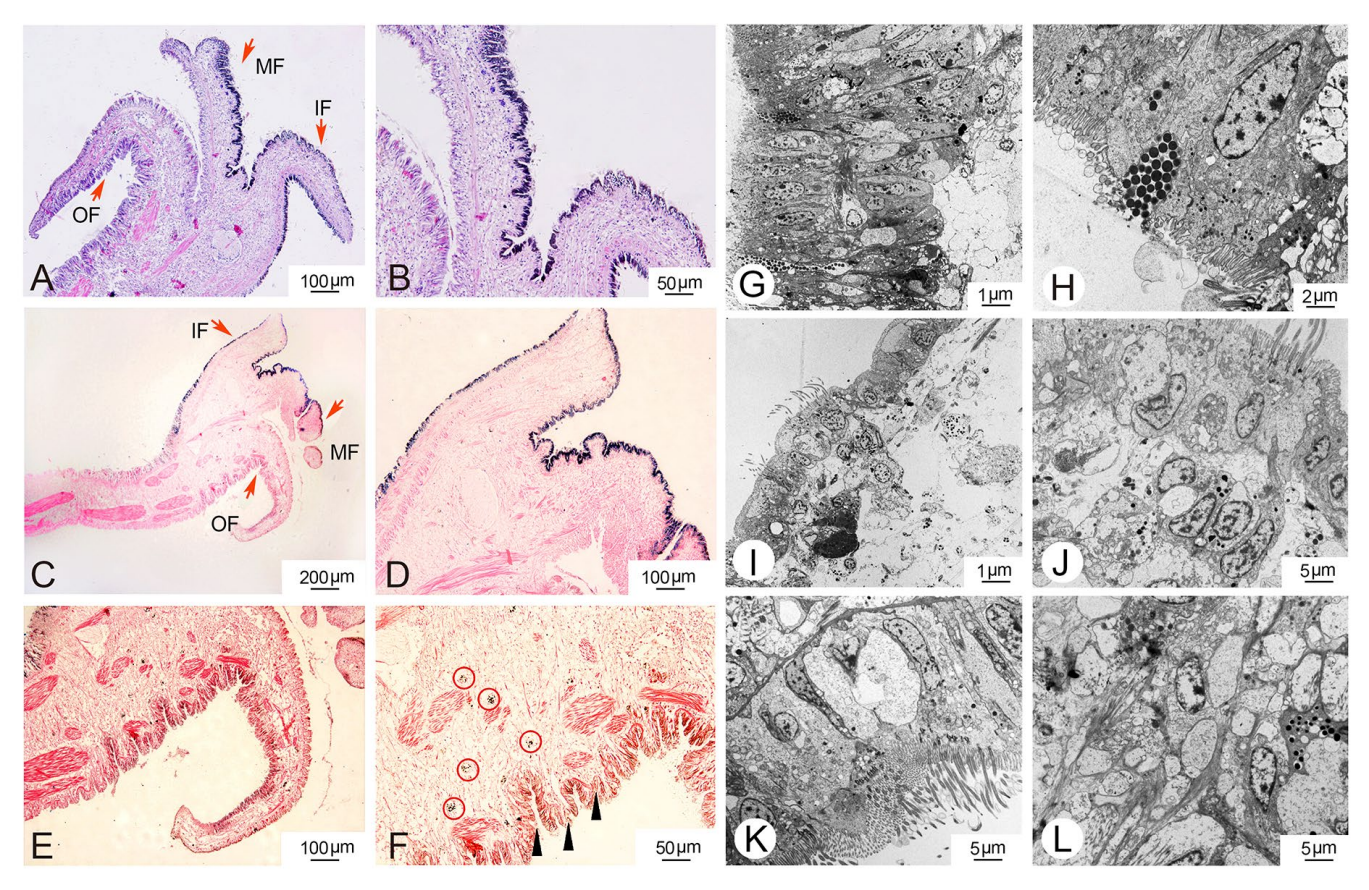
Histological observations were conducted to examine melanin granules in the mantle of *C. gigas*. **A**, **B** Melanin granules in the mantle were visualized using H&E staining. **C**–**F** Ferrous sulfate staining was used to detect melanin granules in the mantle. **G**–**L** Transmission electron microphotographs were taken to observe melanosome structures in the mantle across different folds, including outer fold (**G**, **H**), middle fold (**I**, **J**), and inner fold (**K**, **L**). The arrows indicate different folds of the mantle; red circles represent melanocytes; triangles represent melanin pigmentation

To investigate the distribution of melanin in the mantle, we employed H&E staining, ferrous sulfate staining, and TEM techniques. The mantle may be divided into two regions: the marginal zone and the central zone. The marginal mantle comprises three folds: the outer-, inner- and middle fold. Under light microscopy, pigmented granules were observed in the inner and middle fold of the mantle (Fig. 1A, B), which was consistent with the results of ferrous sulfate staining (Fig. 1C, D). Additionally, the outer fold exhibited lighter pigmentation as observed in the ferrous sulfate staining (Fig. 1E). In the connective tissues of the outer fold, a significant number of vesicles containing melanin granules were observed (Fig. 1F). TEM analysis revealed the ultrastructure of the three distinct folds (Fig. 1G, H, I, J, K, L). Melanin-containing melanosomes were clearly distributed in all three folds. Moreover, a substantial number of melanin-containing granules were observed in trafficking along the outer fold of the mantle epithelium (Fig. 1F).

### Sequence characterization of CgWnt1 and CgWnt2b-a

The amino acid sequences of Wnt1 and Wnt2b-a were obtained from the NCBI database (Supplementary Table S2). A phylogenetic tree was constructed using the neighbor-joining (NJ) method with the Jones Taylor-Thornton (JTT) + Gamma Distributed (G) model in MEGA 7. Motif analysis was conducted using the MEME Suite (https://meme-suite.org/meme/). The analysis of Wnt1 and Wnt2b-a protein sequences from *C. gigas* and other species revealed the presence of similar motifs, as depicted in Supplementary Fig. S1A, B. Phylogenetic analysis of the protein sequences of CgWnt1 and CgWnt2b-a demonstrated a high degree of identity among bivalves. These findings suggest that Wnt1 and Wnt2b-a proteins are well-conserved among invertebrates.

### Expression patterns of *CgWnt1* and *CgWnt2b-a*

**Fig. 2:**
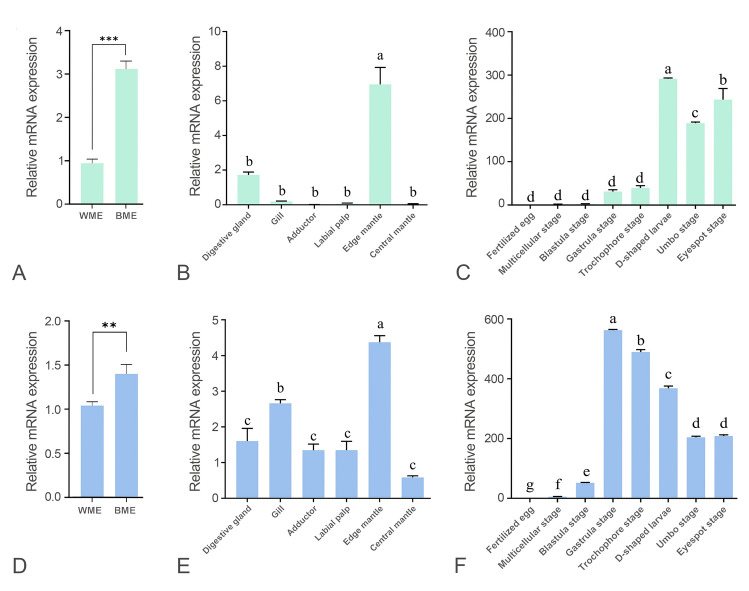
Expression profiles of *CgWnt1* and *CgWnt2b-a*. **A**–**C** Expression characterization of *CgWnt1* in various tissues and different developmental stages, respectively. (**D**–**F**) Expression characterization of *CgWnt2b-a* in various tissues and different developmental stages, respectively. WME refers to the edge mantle of white shell-colored oysters, whereas BME refers to the edge mantle of black shell-colored oysters. Data were presented as means ± SD (n = 3). The significant difference (*P* < 0.05) was indicated by different lowercase letters. *P < 0.05; **P < 0.01; ***P < 0.001

Real-time RT-PCR and ISH techniques were utilized to examine the expression patterns of *CgWnt1* and *CgWnt2b-a*. Overall, expression of *CgWnt1* and *CgWnt2b-a* varied across different tissues. Both genes exhibited higher levels of expression in the edge of the mantle compared to other tissues (Fig. 2B, E). To compare expression profiles between shell color strains, the edge mantle of the black shell color strain and white shell color strain was analyzed. *CgWnt1* and *CgWnt2b-a* showed higher levels of expression in the black shell color oysters than in those with white shell color (*P* < 0.05) (Fig. 2A, D). During different developmental stages, *CgWnt1* expression increased dramatically from the gastrula stage, reaching its peak at the D-shaped larval stage, with lower expression observed before the blastula stage (*P* < 0.05) (Fig. 2C). Conversely, the expression of *CgWnt2b-a* increased rapidly from the blastula larval stage and reached its maximum level at the gastrula stage (Fig. 2F).

**Fig. 3:**
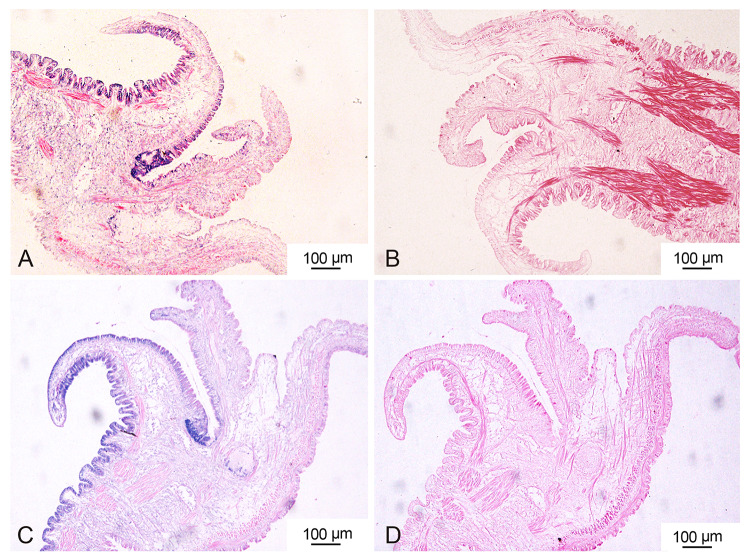
Cellular localization of *CgWnt1* (**A**) and *CgWnt2b-a* (**C**) were determined using anti-sense probes in oyster mantle tissues. Negative controls (**B**, **D**) were detected using sense probes

To determine the spatial pattern of *CgWnt1* and *CgWnt2b-a* expression, ISH was performed. Interestingly, *CgWnt1* and *CgWnt2b-a* were detected specifically in the outer fold of the mantle (Fig. 3).

### RNAi-mediated *CgWnt1 *and *CgWnt2b-a* knockdown in *C. gigas*

**Fig. 4:**
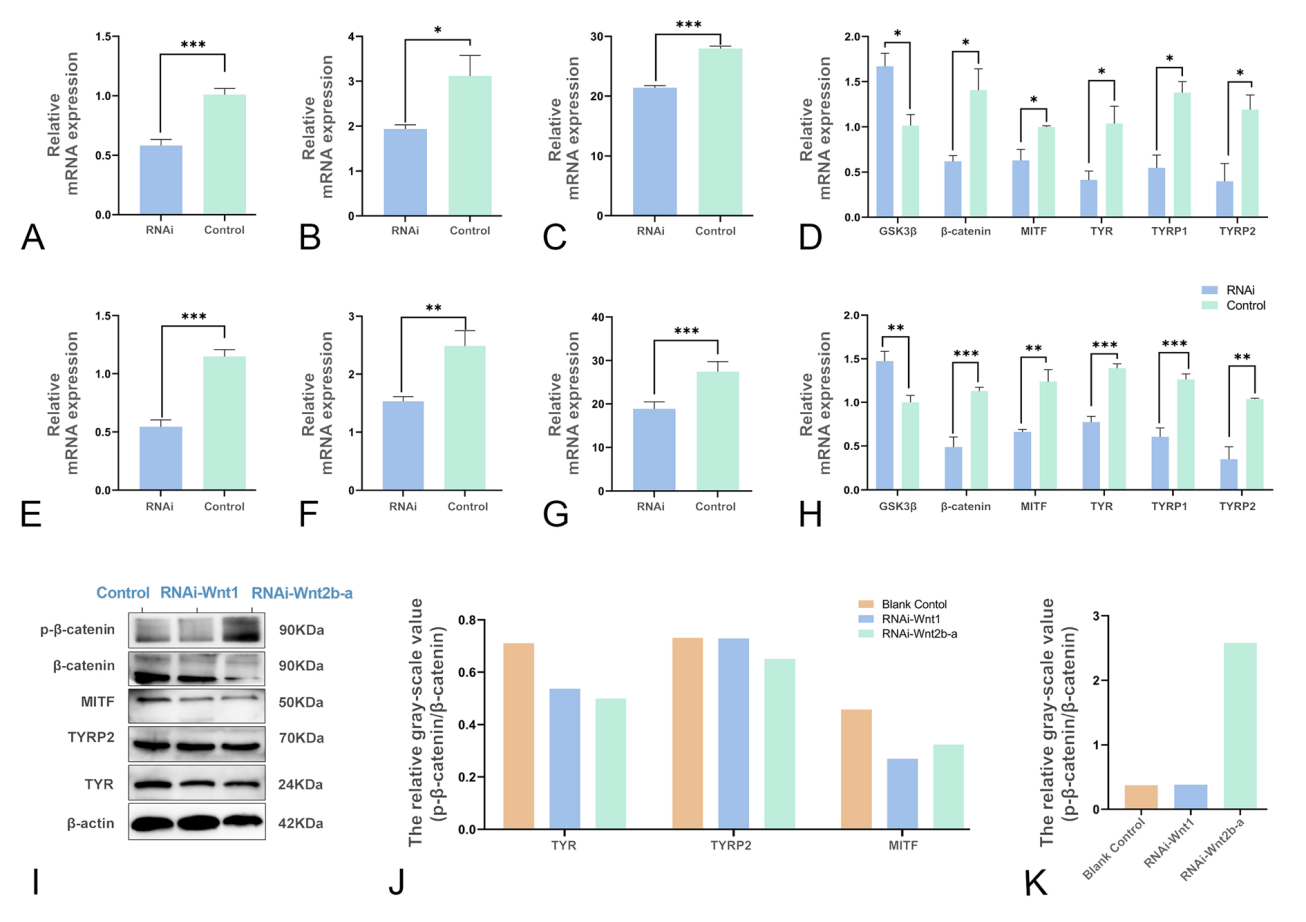
Knockdown of *CgWnt1* and *CgWnt2b-a* inhibits melanogenesis. Expression levels of *CgWnt1* (**A**) and *CgWnt2b-a* (**E**) in the mantle after gene silencing. Tyrosinase activity of *CgWnt1* (**B**) and *CgWnt2b-a* (**F**) in the mantle after gene silencing. Melanin content after silencing *CgWnt1* (**C**) and *CgWnt2b-a* (**G**). Transcriptional levels of downstream genes after *CgWnt1* (**D**) and *CgWnt2b-a* (H) RNAi. (**I**–**K**) Analysis of downstream protein expressions after *CgWnt1* and *CgWnt2b-a* RNAi. Data were presented as means ± SD (n = 3).The significant difference (P < 0.05) was indicated by different lowercase letters. *P < 0.05; **P < 0.01; ***P < 0.001

To assess the role of *CgWnt1* and *CgWnt2b-a* in melanogenesis, RNA interference was conducted in vivo. After one week of RNA interference, the expression levels of *CgWnt1* and *CgWnt2b-a* were reduced by 42.22% and 52.55%, respectively, compared to the control group (Fig. 4A, E). The Wnt downstream genes, including *CgGSK3β*, *CgMITF*, *Cgβ-catenin-like*, *CgTYR*, *CgTYRP1*, and *CgTYRP2*, were examined using qPCR and WB. Except for *CgGSK3β*, the expression levels of the other genes were significantly down-regulated compared to the control group (Fig. 4D, H). Similarly, the protein expression of β-catenin, CgMITF, CgTYR, CgTYRP1, and CgTYRP2 was decreased (Fig. 4I, J, K). However, an increase in the ratio of phosphorylated β-catenin (p-β-catenin) to non-phosphorylated β-catenin was increased, suggesting an inactivation of the Wnt signaling pathway. The tyrosinase activity in the mantles of black shell oysters after RNAi was also measured (Fig. 4B, F). This activity was significantly lower in the *CgWnt1* or *CgWnt2b-a* group compared to the controls (*P* < 0.05). Furthermore, the melanin content decreased considerably by 23.55% and 31.31%, respectively (Fig. 4C, G).

**Fig. 5:**
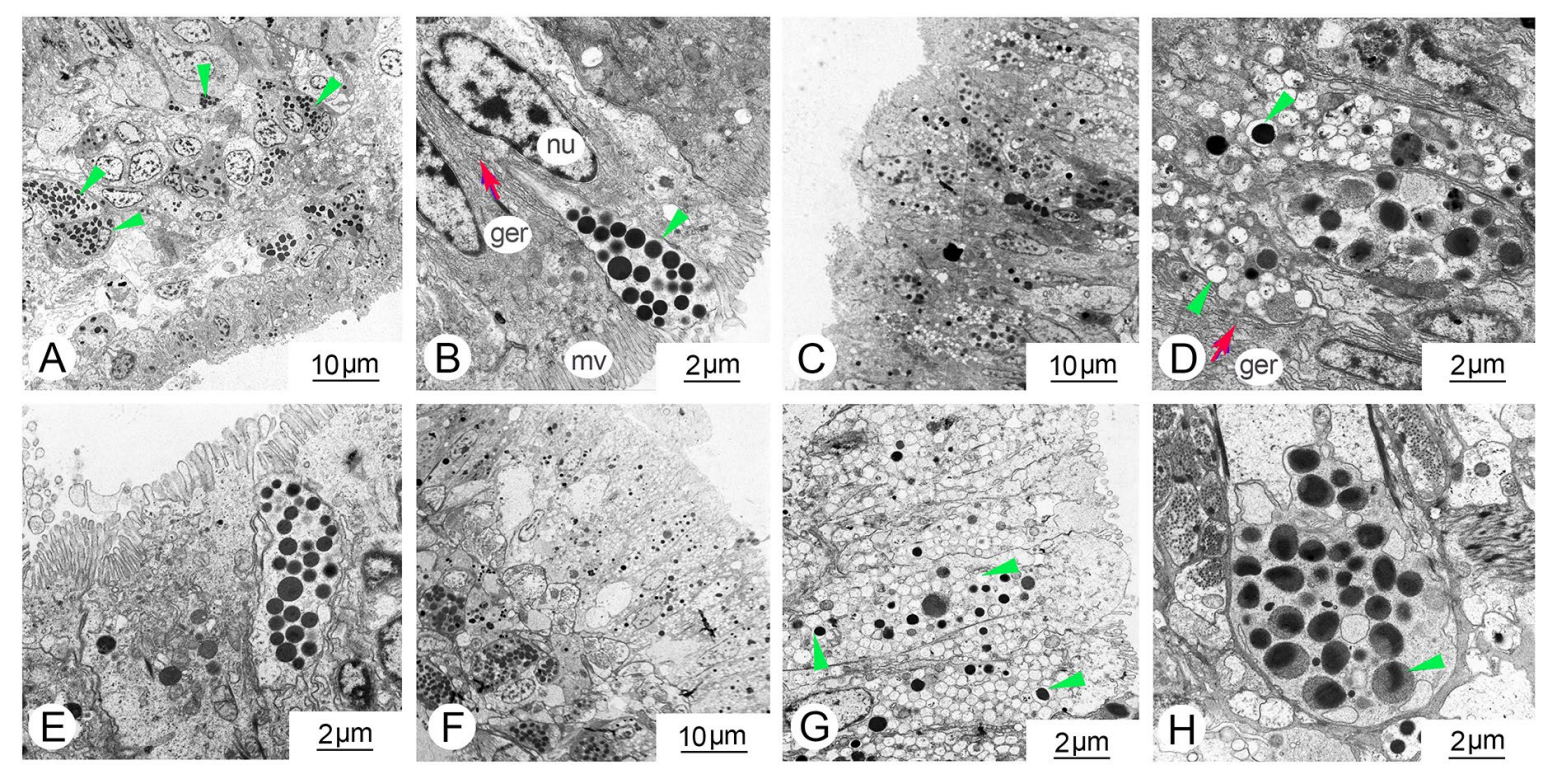
TEM images of *C. gigas* outer fold of the mantle in the (**A**, **B**) control group, (**C**–**E**) *CgWnt1* RNAi group, (**F**–**H**) *CgWnt2b-a* RNAi group. The triangles represent melanosomes. *mv* microvilli; *nu* nucleus; *ger* granular endoplasmic reticulum

The melanogenesis process was examined using transmission electron microscopy. In the control group, a significant quantity of melanosomes was observed in the epithelia of the mantle (Fig. 5). The melanosomes appeared as vesicles with numerous melanin granules. A large number of melanosomes were concentrated towards the lumen of the apical microvillar surface. In contrast to the control group, melanosomes in both the dsWnt1 and dsWnt2b-a RNA interference groups exhibited non-pigmented and partially pigmented characteristics (Fig. 5). Notably, numerous bubble-like melanosomes were observed in the mantle epithelium (Fig. 5).

#### Effects of WIF-1 (Wnt inhibitory factor 1) upregulation on melanogenesis

**Fig. 6:**
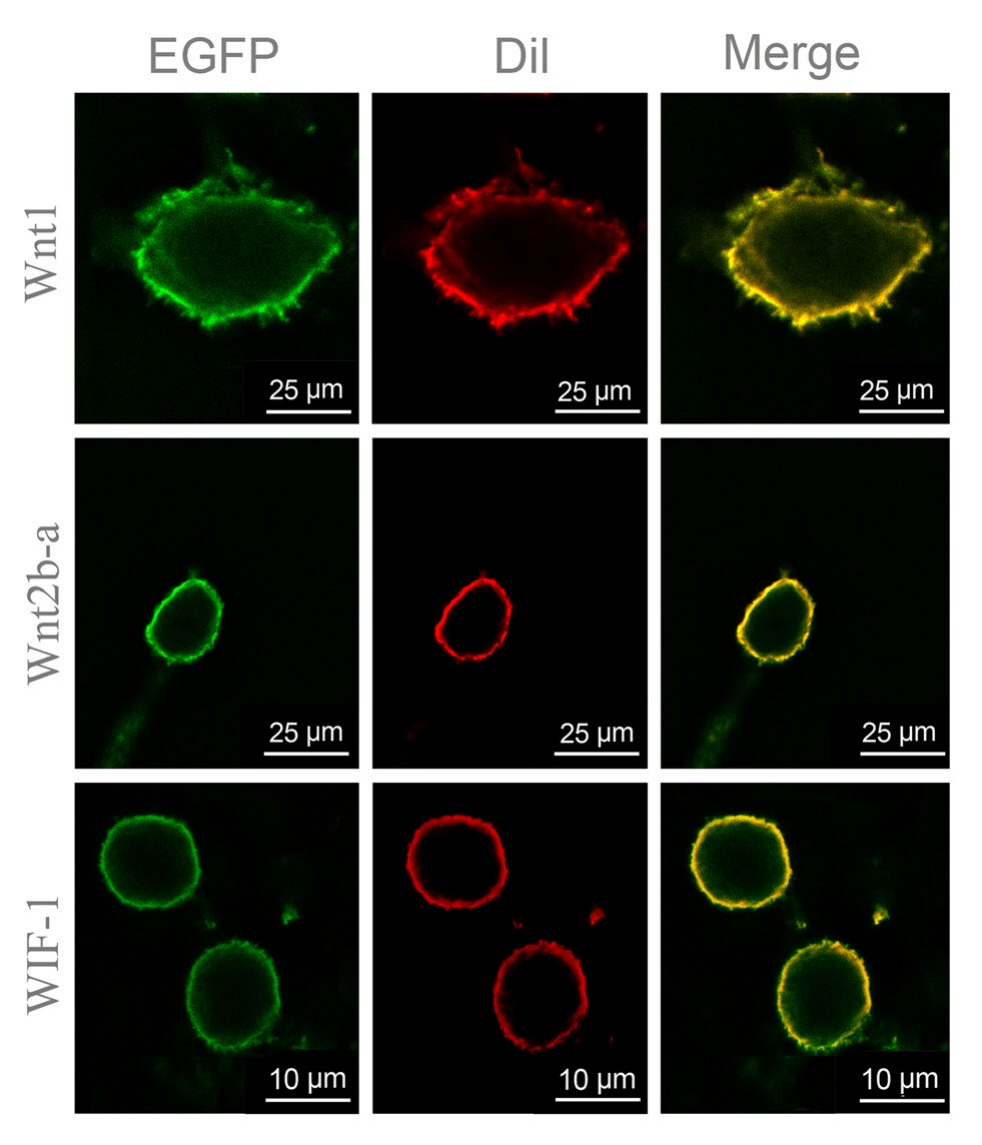
Subcellular localization of Wnt1, Wnt2b-a and WIF-1 proteins in HEK293T cells. Wnt1, Wnt2b-a and WIF-1 were subcloned into pEGFP-N1 and were transiently transfected into 293 T cells, respectively. Dil was the cell membrane fluorescent probe. The expressed Wnt1, Wnt2b-a and WIF-1 protein were colocalized with the Dil probe, and localized to the membrane

**Fig. 7:**
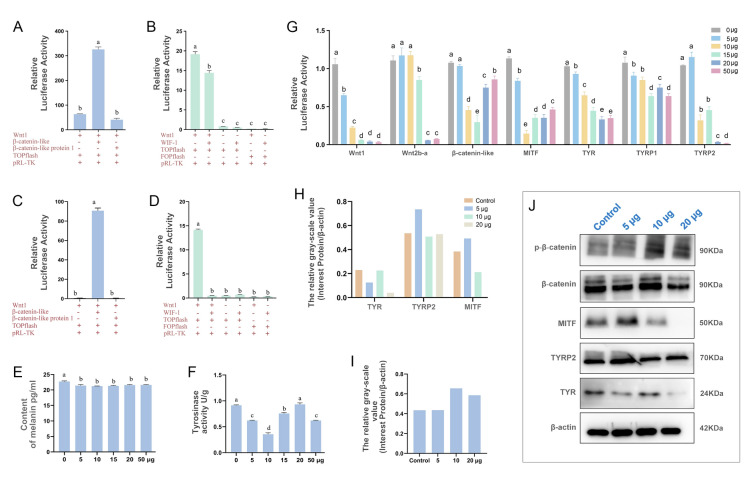
WIF-1 inhibits the Wnt signal transduction pathway. To evaluate the effect of β-catenin/TCF signaling, dual luciferase assays were performed. **A** and **C** Wnt1 and Wnt2b-a bind to β-catenin to activate the Wnt/β-catenin pathway, respectively. **B** and **D** WIF-1 inhibits the Wnt/β-catenin pathway by binding to Wnt1 and Wnt2b-a, respectively. **G** Effect of recombinant protein on the expression of Wnt/β-catenin pathway-related genes. The expression of related genes was measured at 12 h after treatment with recombinant protein WIF-1 at levels of 0, 5, 10, 15, 20, 50 µg/mL. Tyrosinase activity (**E**) and melanin content (**F**) were measured after treatment with recombinant protein. **J** Expression of Wnt/β-catenin pathway-related genes were determined by Western blotting at 12 h after treatment with recombinant protein. Data were presented as means ± SD (n = 3). The significant difference (*P* < 0.05) was indicated by different lowercase letters

Wnt signaling requires β-catenin. Two β-catenin-like genes, Cgβ-catenin-like and Cgβ-catenin-like protein 1, were identified in the oyster genome. To determine whether Cgβ-catenin-like or Cgβ-catenin-like protein 1 could activate the Wnt/β signaling pathway, the CgWnt1 or CgWnt2b-a expression construct was co-transfected with the Cgβ-catenin-like or Cgβ-catenin-like protein 1 expression construct in 293 T cells. The subcellular localization analysis revealed that CgWnt1 and CgWnt2b-a were both located in the cell membrane (Fig. 6). When the 293 T cells reached 70–80% confluence, they were transfected with the TOPflash plasmid, which is a β-catenin-responsive firefly luciferase reporter. The results showed that co-transfection of the CgWnt1 or CgWnt2b-a plasmid with the Cgβ-catenin-like recombinant plasmid significantly increased luciferase activity (Fig. 7A, C), indicating that Cgβ-catenin-like can activate the Wnt/β signaling pathway. Also, the effects of WIF-1 on the Wnt/β-catenin signaling pathway were investigated. The data showed that transfection with the CgWIF-1 plasmid, encoding Wnt inhibitory factor 1, was located in the cell membrane (Fig. 6), and decreased luciferase activity in cells co-transfected with the CgWnt1 or CgWnt2b-a plasmid (Fig. 7B, D). These findings indicate that CgWIF-1 may block Wnt/β-catenin signaling activation.

To assess the inhibitory effects of WIF-1 on Wnt/β-catenin signaling transduction, we analyzed the mRNA expression of *CgWnt1*, *CgWnt2b-a*, *CgMITF*, *Cgβ-catenin-like*, *CgTYR*, *CgTYRP1* and *CgTYRP2* in WIF-1 treated cells. The results demonstrated a dose-dependent inhibition of their expression (Fig. 7G). Western blot analysis further confirmed the decreased expression of TYR, TYRP2, and MITF at the protein levels. As expected, β-catenin phosphorylation was significantly increased in the WIF-1 treated cells (Fig. 7H, I, J). Additionally, we assessed the tyrosinase activity and melanin content after WIF-1 treatment. The results indicated that both the tyrosinase activity and the melanin content were significantly decreased in response to WIF-1 treatment at concentrations of 10 μg/mL and 5 μg/mL, respectively (Fig. 7E, F).

### Salinomycin modulates the Wnt/β-catenin pathway

**Fig. 8:**
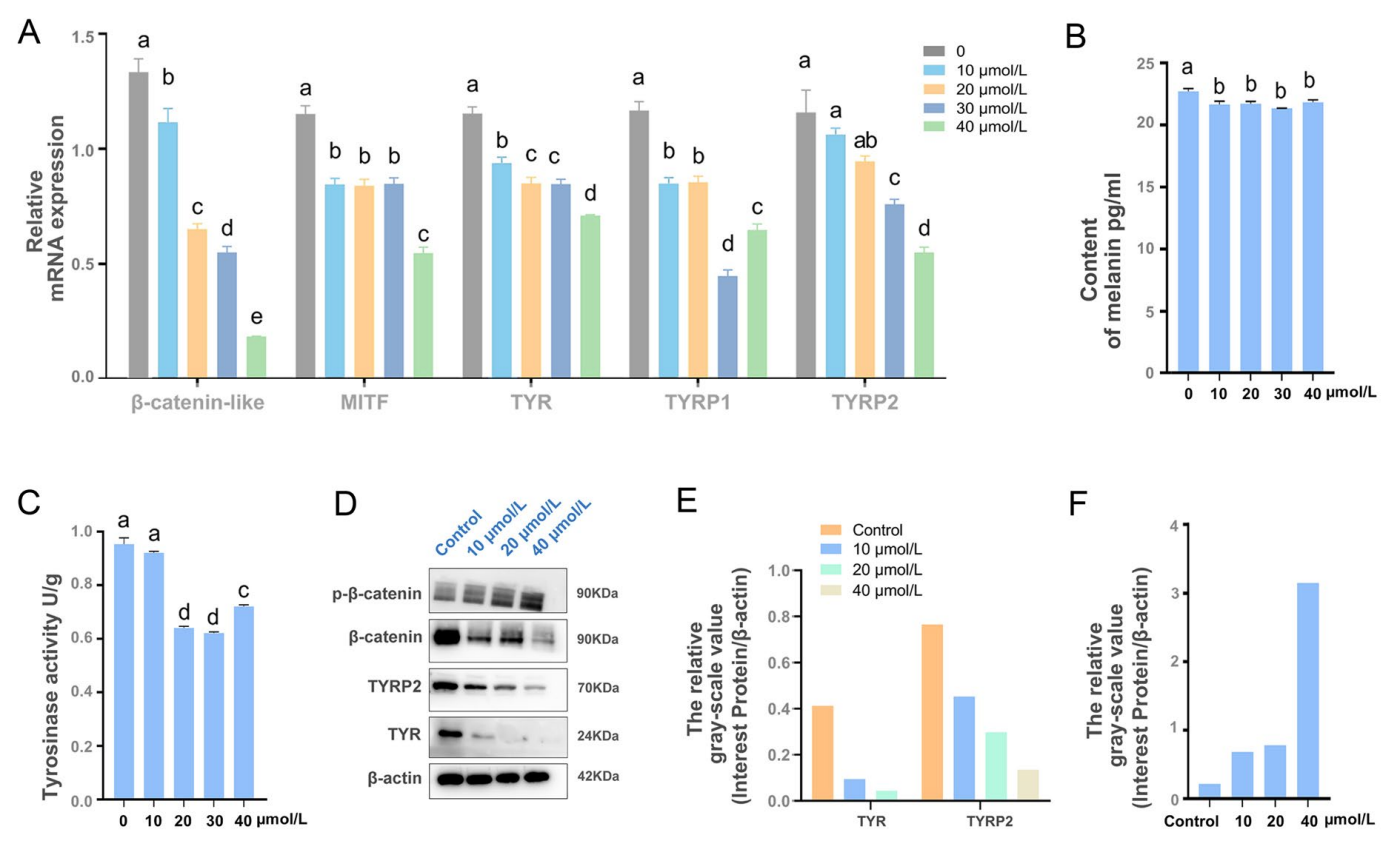
Salinomycin inhibits the Wnt/β-catenin signaling pathway. Expression of Wnt/β-catenin signaling pathway-related genes was measured at 12 h after treatment with salinomycin at levels of 0, 10, 20, 30, and 40 µmol/L using real-time PCR (**A**) and Western blotting (**D**). Tyrosinase activity (**B**) and melanin content (**C**) were measured after treatment. Data were presented as means ± SD (n = 3). The significant difference (*P* < 0.05) was indicated by different lowercase letters

To further investigate the effect of Wnt/β-catenin pathway inhibition on tyrosinase activity in mantle cells, a primary cell culture of mantle was treated with a Wnt signal inhibitor, salinomycin, at different concentrations. Salinomycin treatment significantly decreased the mRNA levels of *CgMITF*, *Cgβ-catenin-like*, *CgTYR*, *CgTYRP1* and *CgTYRP2* (*P* < 0.05) (Fig. 8A). Their protein expression was consistent with the qPCR results (Fig. 8D, E, F). In addition, the phosphorylation of β-catenin protein increased dramatically after treatment. Moreover, the tyrosinase activity of primary mantle cells was significantly decreased in response to the Wnt signal inhibitor (salinomycin) at a concentration of 20 μmol/L (Fig. 8B). Additionally, the melanin content in cultured mantle cells was significantly decreased at a concentration of 10 μmol/L (Fig. 8C), providing further support for the involvement of Wnt/β-catenin signaling in melanogenesis.

## Discussion

Mollusca shells exhibit a wide range of colors, which are directly deposited by epithelial cells in the mantle tissue. In this study, we performed histological analysis of melanin synthesis in mantle tissues. In the case of *C. gigas*, the marginal mantle typically consists of three folds: an outer fold for secretion, a middle fold for sensing, and an inner fold for muscular movement (Audino et al. 2015). We found that melanin granules are primarily deposited in the inner fold and inner surface of the middle fold, similar to observations in *Pinctada maritlza* (Jabbour-Zahab et al. 1992). However, the outer fold of the mantle epithelium exhibited light pigmentation, and numerous melanocytes were discovered in the connective tissues using ferrous sulfate staining. This indicates that melanocytes were transported to the epithelium of the outer fold and melanin was secreted there. Our studies further extended previous findings that the periostracum, which is responsible for shell pigmentation, is synthesized in the periostracal groove located between the outer and middle folds. Pigments are deposited on a tanned organic membrane that covers the inner calcified shell layer (Bubel 1973).

Multiple investigations have demonstrated that genes involved in melanin synthesis are expressed in the outer fold (Min et al. 2022b; Zhu et al. 2021, 2022) suggesting that they may play a role in melanocyte development and maturation. Similar to observations in fish melanophores, it has been reported that melanosomes may move along microtubule tracks (Nascimento et al. 2003). In bivalves, muscle fibers traverse the inner and outer surfaces of mantle tissues, suggesting the possibility of melanin transfer from the inner and middle folds to the outer folds directly through actin filaments (Zhu et al. 2023). Furthermore, the mechanism by which matured melanosomes are trafficked to the shell is not well understood. In vertebrates, four models of melanosomes transport have been proposed. These include keratinocyte cytophagocytosis of dendrites or filopodia tips, direct transfer across a membrane channel, shedding of vesicles followed by keratinocyte engulfment, and exocytosis-mediated secretion of naked melanin (Tian et al. 2021b).

In our study, TEM observations revealed the presence of melanosomes in all three folds, with a substantial abundance of melanin-containing granules trafficking along the outer fold of the mantle epithelium and being transferred via exocytosis-mediated secretion. It has been reported that melanosomes in the outer fold exhibit higher melanotic activity compared to the other two folds (Zhu et al. 2023). Moreover, there are four distinct stages involved in the development and maturation of melanosomes in vertebrates. Stages I–II represent the unpigmented phases, referred to as premelanosomes, during which the matrix is formed in preparation for melanin deposition. Melanin deposition is initiated at stage III and completed at stage IV. During stages III–IV, genes associated with melanogenesis appear to play crucial roles (Wiriyasermkul et al. 2020). These findings support the hypothesis that melanosomes originate in the middle and inner folds of the mantle tissues, are transported to the outer fold via actin-dependent filaments, undergo maturation with the assistance of various genes, and are subsequently released to the shell through exocytosis.

Melanogenesis is regulated through complex receptor-dependent and -independent mechanisms (Slominski et al. 2004). The Wnt/β-catenin pathway plays a crucial role in melanocyte differentiation and development. Melanosome biogenesis, formation, and maturation may be classified into four distinct morphologically characterized stages (Wiriyasermkul et al. 2020). Melanosomes lack melanin production in stages I-II and undergo maturation in stages III-IV due to the activities of several genes encoding melanogenic enzymes and iron transport proteins (Aspengren et al. 2009). In vertebrates, Wnt1 and Wnt2b have been shown to be involved in melanin production (Sun et al. 2023; Yang et al. 2018). Our studies revealed higher expression of Wnt1 and Wnt2b-a in the mantle tissues of the black phenotype compared to the white phenotype. Epigenetic mechanisms enable variations in gene expression, where the N-terminal domain of Wnt undergoes palmitoleic acid modification, facilitating recognition by the receptor Frizzled (Rim et al. 2022). In the black and white phenotype of *C. gigas*, it is likely that the palmitoleic acid modification level of Wnt differs, thereby influencing the signal transduction and downstream gene activation to some extent. Additionally, our study showed that the melanosomes in the control group exhibited numerous and mature ellipsoidal-shaped structures with a high melanotic content in the outer fold of the epithelium. In contrast, the melanosomes in the dsWnt1 and dsWnt2b-a interference groups displayed reduced density and an increased presence of vacuoles, indicating poor melanogenesis. Consistent with our findings, the injection of Wnt3a and Wnt10b mRNA stimulated melanocyte differentiation in mice, whereas Wnt inhibitors injection had the opposite effect (Guo et al. 2016; Ye et al. 2013).

Also, we demonstrated that Wnt1 and Wnt2b-a, members of the canonical Wnt family, may activate luciferase activity in the presence of β-catenin-like protein in *C. gigas*. This is consistent with the notion that Wnts regulate gene transcription by inhibiting the cytoplasmic degradation of β-catenin and interacting with the protein complex in the nucleus (Dunn et al. 2005; Liu et al. 2002; Rim et al. 2022; Shang et al. 2017; van Noort et al. 2002). Stabilized β-catenin interacts with members of the LEF/TCF transcription factors to modulate MITF transcription (Schepsky et al. 2006). MITF serves as the principal regulator of melanocyte differentiation, survival, and proliferation by controlling numerous genes, including those encoding melanogenic enzymes such as TYR, TYRP1, and TYRP2 (Levy et al. 2006). The down-regulation of MITF, TYR, TYRP1, and TYRP2 observed in our study, as a result of Wnt1 and Wnt2b-a interference, contributed to decreased melanin production. Collectively, Wnt ligands represent the main source of Wnt activation in melanocytes, and their absence cannot be compensated by other sources. The Wnt signaling pathway may also be regulated by extracellular antagonists/inhibitors.

The extracellular antagonists of the Wnt signaling pathway may be categorized into two functional groups: the sFRP (secreted Frizzled-related protein) class and the Dkk (Dickkopf) class (Kawano and Kypta 2003). Both types of molecules, albeit in different ways, block ligand-receptor interactions. WIF-1, a member of the sFRP family that directly binds to Wnts, has lost its capacity to bind to the Wnt receptor complex (Cruciat and Niehrs 2013). It has been reported that the down-regulation of WIF-1 is expected to induce hyperpigmentation of the skin (Kim et al. 2013a, b). In fact, the mRNA levels of WIF-1 are significantly lower in black shell-colored oysters compared to white shelled specimens (Supplementary Fig. S3). Therefore, we hypothesized that the up-regulation of WIF-1 in the mantle of *C. gigas* may have a negative impact on melanogenesis.

Our results showed that the overexpression of WIF-1 through treatment with recombinant CgWIF-1 protein exerted its action on canonical pathways, leading to a decrease in the expression levels of β-catenin and genes encoding melanogenic enzymes. Also, it stimulated a decrease in tyrosinase activity and the content of melanin. Additionally, the dual luciferase reporter experiment revealed a significant suppression of luciferase activity when co-transfected with WIF-1, suggesting that this is likely to interact with Wnts blocking ligand-receptor interactions. Moreover, WIF-1 knockdown promoted NFATc2 dephosphorylation and nuclear translocation, indicating that the noncanonical route stimulates target gene transcription (Kim et al. 2013a, b). Consequently, sFRP antagonists inhibit both canonical and noncanonical pathways, thereby affecting pigmentation.

Salinomycin is a potent inhibitor of the Wnt/β-catenin signaling pathway, similar to Dkk class antagonists, as it inhibits the proper functioning of the co-receptor LRP5/6 (Cruciat and Niehrs 2013). Salinomycin has been shown to block Wnt-induced LRP6 phosphorylation and degradation (Lu et al. 2011). Activation of the Wnt pathway promotes the phosphorylation of the co-receptor LRP5/6, leading to a phosphorylated motif that inhibits GSK3β. This, in turn, stabilizes β-catenin and increases the transcription of β-catenin target genes (MacDonald and He 2012). In our investigation, primary cells from mantle tissues were treated with various doses of salinomycin, and the findings indicated that the transcription of β-catenin and its target genes was repressed. These results suggest that inhibiting the co-receptor phosphorylation of the Wnt/β-catenin pathway hinders the cascade of downstream signaling transduction.

**Fig. 9:**
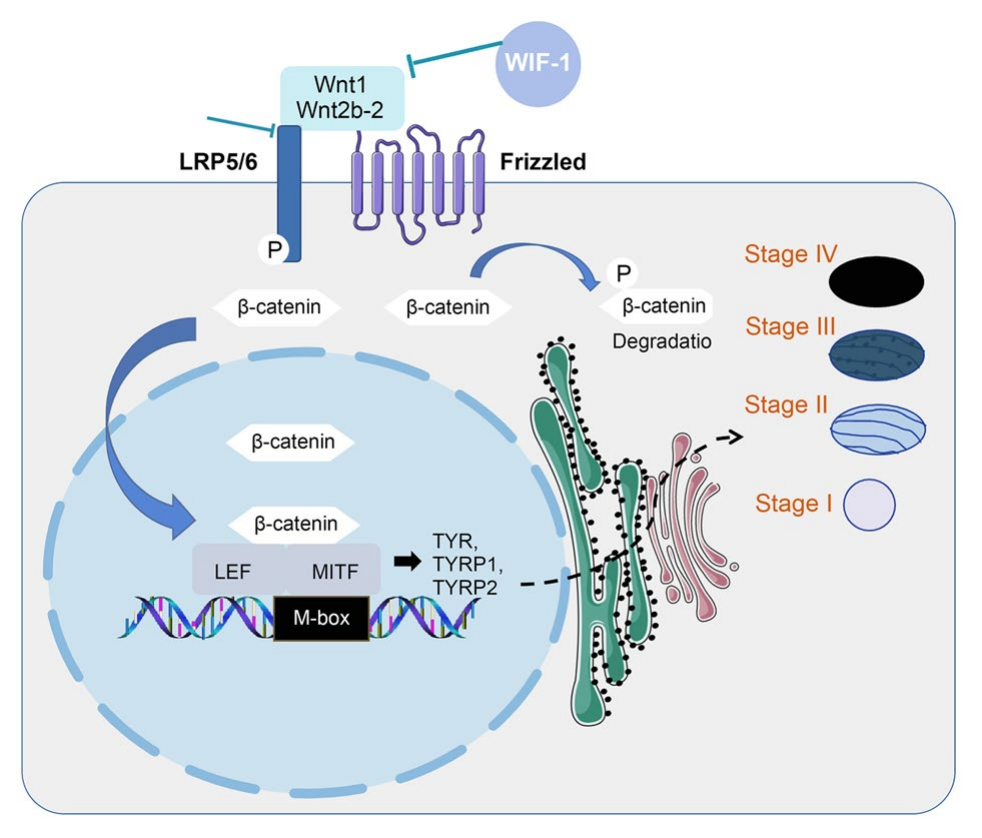
Diagram of putative gene pathway in the *C. gigas* shell pigmentation process. The role of MITF in the melanogenesis cascade in *C. gigas* is depicted in the diagram. MITF plays a dominant role in the development and maturation of melanosomes, which is followed by melanogenesis. The melanogenesis process involves continuous enzymatic protein reactions, including TYR, TYRP1, and TYRP2, which contribute to the synthesis and storage of melanin pigments in melanosomes. Melanosome formation and maturation consist of four stages. Stages I and II represent the nonpigmented stages known as premelanosomes, where the matrix is formed for melanin deposition. The melanogenesis pathway initiates at stage III and completes at stage IV

In conclusion, our results demonstrate that the inhibition of the Wnt/β-catenin pathway effectively blocks melanogenesis in *C. gigas*. The reduction in melanin production may be attributed to the downregulation of both MITF and its downstream target gene expression (Fig. 9). Additionally, we found that melanin-containing melanosomes were distributed in all three folds, originating from the middle and inner folds and further maturing in the outer fold. Our research has elucidated the potential melanin pigmentation process in the mantle fold of *C. gigas*, underscoring the modularity function of the mantle tissues. However, the precise regulatory mechanism governing folds in pigmentation needs to be investigated in future studies to shed light on the targeted shell color breeding.

### Supplementary Information

Below is the link to the electronic supplementary material.

Supplementary file1 (DOC 5588 KB)

Supplementary file2 (DOC 7431 KB)

Supplementary file3 (DOC 216 KB)

Supplementary file4 (DOCX 33 KB)

Supplementary file5 (DOCX 15 KB)

## Data Availability

The data that supports the findings of this study are included in this published article and its supplementary information file.
