# Supplementary material for: Examination of wnt signaling mediated melanin transport and shell color formation in Pacific oyster (Crassostrea gigas)
Source: Mar Life Sci Technol. 2024 Jun 6;6(3):488–501. doi: 10.1007/s42995-024-00221-5 (PMC11358575; doi:10.1007/s42995-024-00221-5)
Supplement: Supplementary file 1 — Supplementary file1 (DOC 5588 KB) [file 42995_2024_221_MOESM1_ESM.doc]

**Supplementary Fig S1.**Phylogenetic and moitf analysis of *Wnt1* and *Wnt2b-a*. (A)Phylogenetic and moitf analysis of *Wnt1*. (B)Phylogenetic and moitf analysis of *Wnt2b-a*.

**
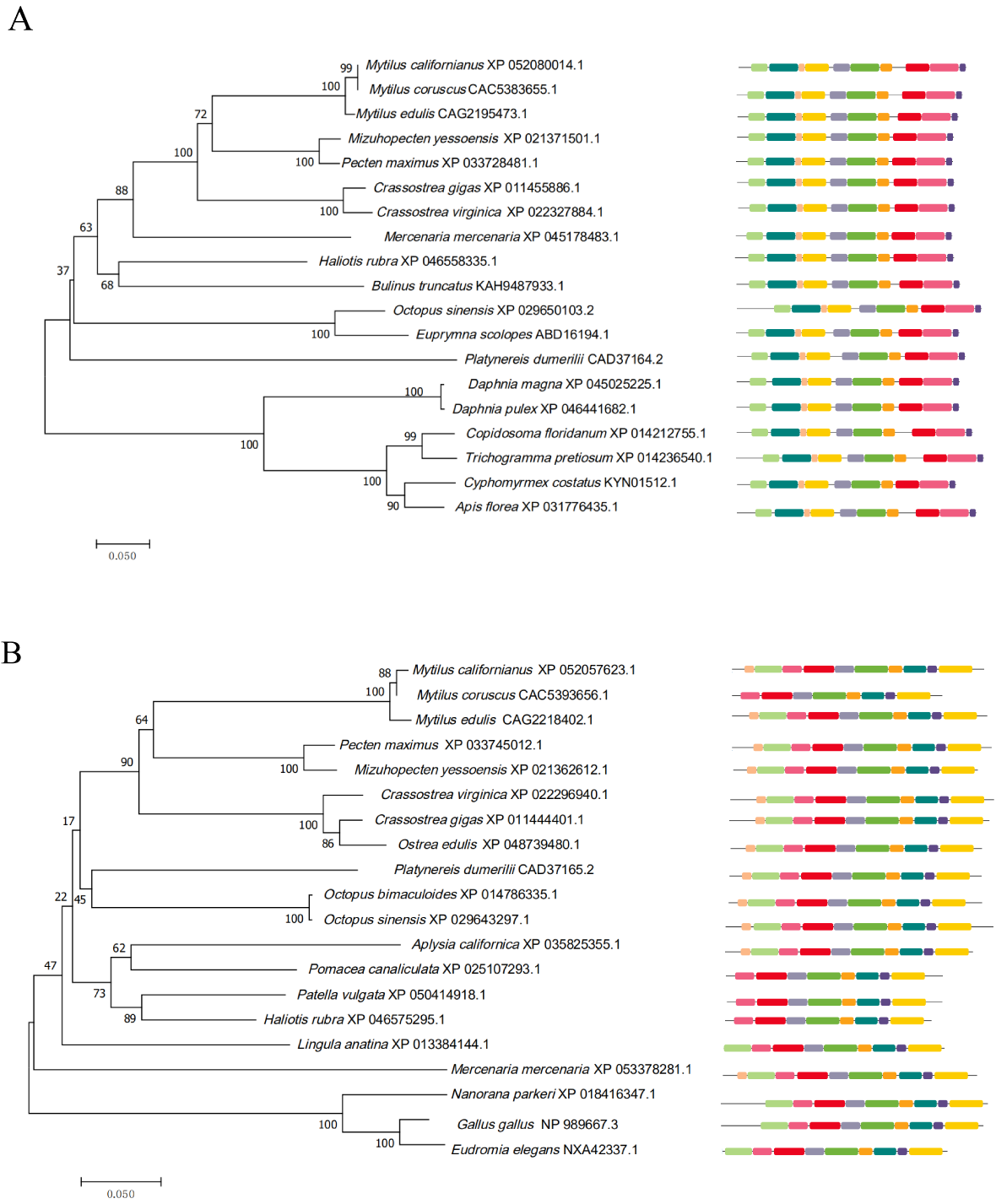
**
