# Supplementary material for: Examination of wnt signaling mediated melanin transport and shell color formation in Pacific oyster (Crassostrea gigas)
Source: Mar Life Sci Technol. 2024 Jun 6;6(3):488–501. doi: 10.1007/s42995-024-00221-5 (PMC11358575; doi:10.1007/s42995-024-00221-5)
Supplement: Supplementary file 2 — Supplementary file2 (DOC 7431 KB) [file 42995_2024_221_MOESM2_ESM.doc]

**Supplementary Fig S2.**

Production of recombinant protein CgWIF. (A)The recombinant protein was induced by IPTG with final concentration of 1 mmol/L at different temperature for 12h. (B)The recombinant protein was induced by IPTG with final concentration of 1 mM at 37 ℃ for different periods. (C) The purification of the the recombinant protein CgWIF, which was induced by IPTG with final concentration of 1 mM at 37 ℃ for 4h.

**
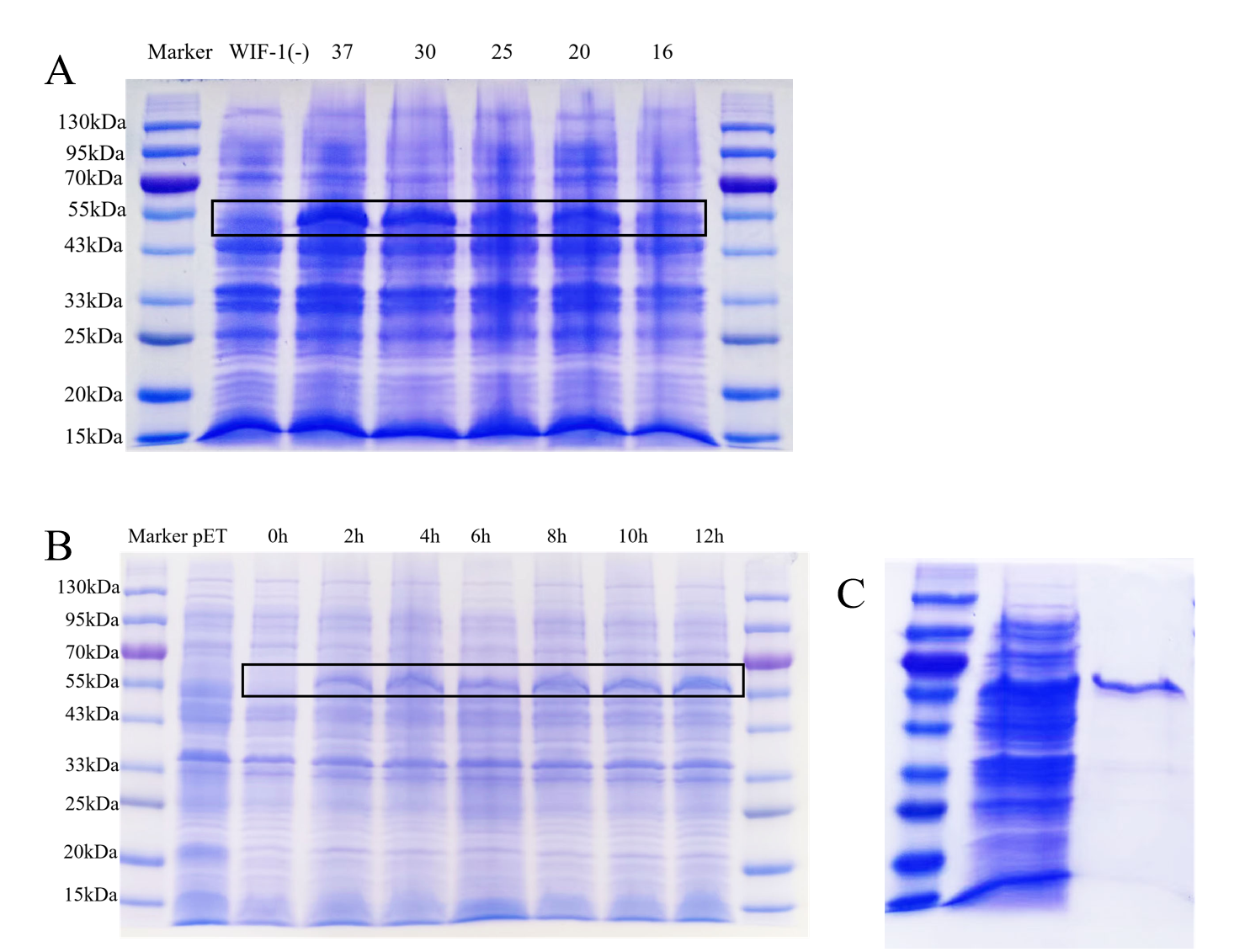
**
