# Supplementary material for: Examination of wnt signaling mediated melanin transport and shell color formation in Pacific oyster (Crassostrea gigas)
Source: Mar Life Sci Technol. 2024 Jun 6;6(3):488–501. doi: 10.1007/s42995-024-00221-5 (PMC11358575; doi:10.1007/s42995-024-00221-5)

**Supplementary Fig S3.**

The relative expression of *CgWIF-1* between the edge mantle of black shell color oyster and white shell color oyster. Data were presented as means±SD (n = 3). The significant difference (*P*<0.05) was presented by different lowercase letters. *: *P* < 0.05; **: *P* < 0.01; ***: *P* < 0.001.


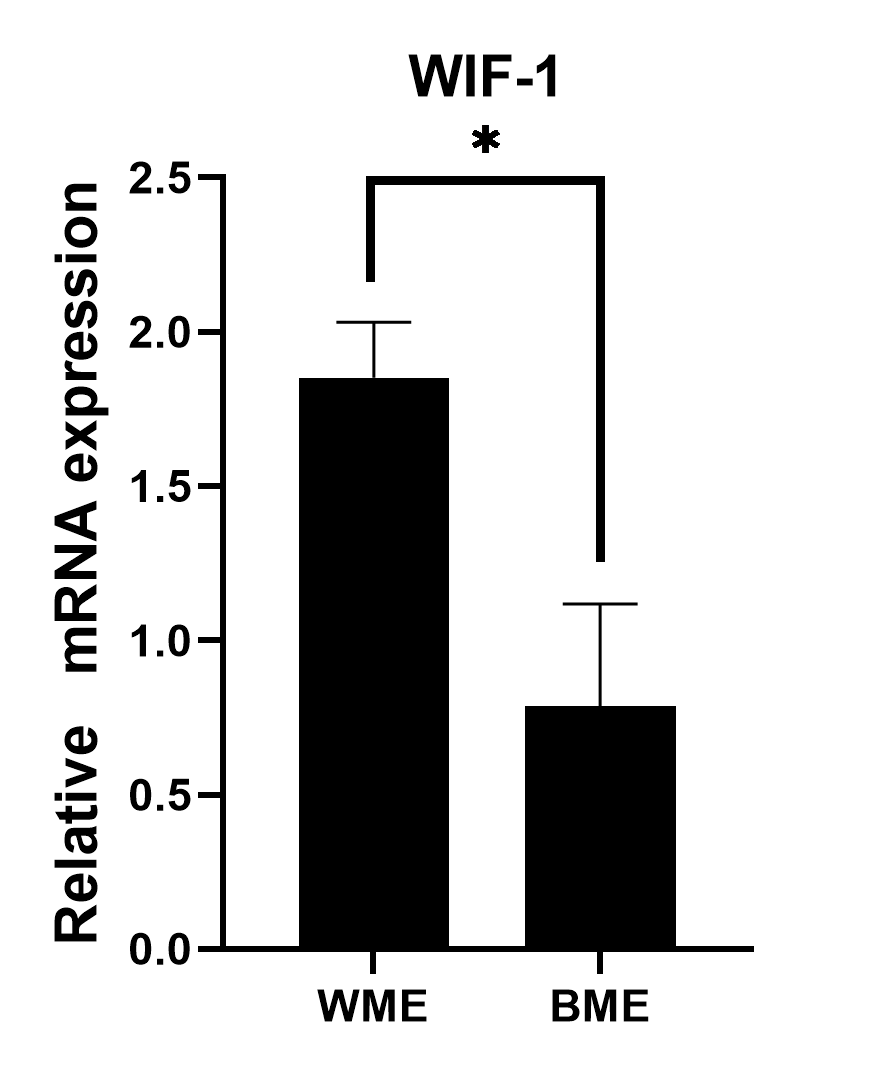

Supplement: Supplementary file 3 — Supplementary file3 (DOC 216 KB) [file 42995_2024_221_MOESM3_ESM.doc]
