# Supplementary material for: Examination of wnt signaling mediated melanin transport and shell color formation in Pacific oyster (Crassostrea gigas)
Source: Mar Life Sci Technol. 2024 Jun 6;6(3):488–501. doi: 10.1007/s42995-024-00221-5 (PMC11358575; doi:10.1007/s42995-024-00221-5)
Supplement: Supplementary file 4 — Supplementary file4 (DOCX 33 KB) [file 42995_2024_221_MOESM4_ESM.docx]

**Supplementary Table S1.** Primers used in this study. The lower case letters were the T7 promoter sequence, used to synthesis sense, antisense probe, and double-stranded RNA. The underlined letters were homologous arm, contained 15-20 bases that were homologous to one end of the DNA fragments to which it will be joined, which were used to construct plasmids.

| **Purpose** | **Primer name** | **Primer sequence (5**' **to 3**'**)** |
| --- | --- | --- |
| Quantitative RT-PCR | Wnt1-F | TACATAACAACGAAGCTGGAAGA |
|  | Wnt1-R | CTGTCCTTGAGCCTGTCCC |
|  | Wnt2b-a-F | CTGGTTTGAGTGGGGAGGA |
|  | Wnt2b-a-R | GGCACGGTTGTTATGGAGATT |
|  | GSK3β-F | AATAAGGATGGCAGCAAGATAAC |
|  | GSK3β-R | TGATAAACAACCCCAAAAGACC |
|  | β-catenin-like-F | CAGAGGTTATGAGCACCAGATTC |
|  | Βcatenin-like-R | AGGTCAGGCAGGTTTTCCA |
|  | TYR-F | GTACGATTCTTGTGGTCGGC |
|  | TYR-R | GAGGTGAAGCGTCATCCAAAG |
|  | TYRP1-F | CGAGGCGTTTCCAGTTTGTG |
|  | TYRP1-R | TGGCAGTAGCCGGTGAATTT |
|  | TYRP2-F | TCGTCGATGAAAGGCAACCA |
|  | TYRP2-R | CATACACTGGACAAGCGGGT |
|  | MITF-F | TGCAGCAAATGGAGTTGGTC |
|  | MITF-R | TGGAATTTGGCGGCCTGAAA |
|  | EF1**α**-F | AGTCACCAAGGCTGCACAGAAAG |
|  | EF1**α**-R | TCCGACGTATTTCTTTGCGATGT |
|  | ARF1-F | TCAGGACAAGATCCGACCACTGT |
|  | ARF1-R | GCAGCGTCTCTAAGTTCATCCTCATTA |
|  | GAPDH-F | AGAACATCGTCAGCAACGCATCC |
|  | GAPDH-R | CCTCTACCACCACGCCAATCCT |
| ISH | Wnt1-sense-probe-F | gatcactaatacgactcactataggg TGGAAAAGACTGGGAGTGGG |
|  | Wnt1-sense-probe-R | TTCACAGTACGAAGGAGACGA |
|  | Wnt1-antisense-probe-F | TGGAAAAGACTGGGAGTGGG |
|  | Wnt1-antisense-probe-R | gatcactaatacgactcactatagggTTCACAGTACGAAGGAGACGA |
|  | Wnt2b-a-sense-probe-F | gatcactaatacgactcactataggg ATCAAGGAATGCCAGAGCC |
|  | Wnt2b-a-sense-probe-R | GCCGTTGTACTTCCGCTTC |
|  | Wnt2b-a-antisense-probe-F | ATCAAGGAATGCCAGAGCC |
|  | Wnt2b-a-antisense-probe-R | gatcactaatacgactcactatagggGCCGTTGTACTTCCGCTTC |
| dsRNA synthesis | Wnt1-dsRNA-F | taatacgactcactatagggCAGTCCAAGCCTTTTACCGT |
|  | Wnt1-dsRNA-R | taatacgactcactatagggCTGTCCTTGAGCCTGTCCC |
|  | Wnt2b-a-dsRNA-F | taatacgactcactatagggATCAAGGAATGCCAGAGCC |
|  | Wnt2b-a-dsRNA-R | taatacgactcactatagggCCGCTTCAGGTATTCTCCAAC |
| Expression plasmid | pcDNA3.1-Wnt1-F | TAGTCCAGTGTGGTGGAATTCATGCGGACTCTGAATAGTTTATTG |
|  | pcDNA3.1-Wnt1-R | AACGGGCCCTCTAGACTCGAGCTATTTACACGTGTGGAGTACTTTT |
|  | pcDNA3.1-Wnt2b-a -F | TAGTCCAGTGTGGTGGAATTCATGACAAATGTACGATGCCGT |
|  | pcDNA3.1- Wnt2b-a -R | AACGGGCCCTCTAGACTCGAGTTATCCATTCAGAGAATTGCCA |
|  | pcDNA3.1-WIF -F | gccggtaccgagctcggatccAtgtgtaaagcaaaactgcaggt |
|  | pcDNA3.1- WIF -R | tgctggatatctgcagaattcCccagggcttcataaaagttct |
|  | pcDNA3.1-β-catenin-like -F | TAGTCCAGTGTGGTGGAATTCATGAGTACATATCAGATGAACCAGA |
|  | pcDNA3.1-β-catenin-like -R | AACGGGCCCTCTAGACTCGAGTTACAAGTCGGTGTCAAACCA |
|  | pcDNA3.1-β-catenin-like protein 1 -F | TAGTCCAGTGTGGTGGAATTCATGGACGTCGGGGAGCTA |
|  | pcDNA3.1-β-catenin-like protein 1-R | AACGGGCCCTCTAGACTCGAGTCACAAAAACTTGTCCACTAACTGC |
| Recombinant protein plasmid | WIF-pET32a-F | gccatggctgatatcggatccCGCAGCCGCACCCGTTTG |
|  | WIF-pET32a-R | ttgtcgacggagctcgaattcGCGCACAAACGGTTTGCG |
| Subcellular  location plasmid | Wnt1-pEGFP-F | TcagatctcgagctcaagcttATGCGGACTCTGAATAGTTTATTG |
|  | Wnt1-pEGFP-R | atggtggcgaccggtggatccTTTACACGTGTGGAGTACTTTT |
|  | Wnt2b-a-pEGFP-F | TcagatctcgagctcaagcttATGACAAATGTACGATGCCGT |
|  | Wnt2b-a-pEGFP-R | atggtggcgaccggtggatccTCCATTCAGAGAATTGCCA |
|  | WIF-pEGFP-F | TcagatctcgagctcaagcttATGTGTAAAGCAAAACTGCAGGT |
|  | WIF-pEGFP-R | atggtggcgaccggtggatccCCTCACAAAAGGTTTCCG |

Note: F indicates forward primer and R indicates reverse primer.
