# Supplementary material for: Examination of wnt signaling mediated melanin transport and shell color formation in Pacific oyster (Crassostrea gigas)
Source: Mar Life Sci Technol. 2024 Jun 6;6(3):488–501. doi: 10.1007/s42995-024-00221-5 (PMC11358575; doi:10.1007/s42995-024-00221-5)
Supplement: Supplementary file 5 — Supplementary file5 (DOCX 15 KB) [file 42995_2024_221_MOESM5_ESM.docx]

**Supplementary Table S2.**The accession number of Wnt1,and Wnt2b-a protein sequences used in phylogenetic.

| Species | protein | Accession number |
| --- | --- | --- |
| Crassostrea gigas | Wnt-1 | XP_011455886.1 |
| Crassostrea virginica | Wnt-1-like | XP_022327884.1 |
| Mizuhopecten yessoensis | Wnt-1-like | XP_021371501.1 |
| Mytilus edulis | Wnt-1 | CAG2195473.1 |
| Mytilus californianus | Wnt-1-like | XP_052080014.1 |
| Mytilus coruscus | Wnt-1 | CAC5383655.1 |
| Pecten maximus | Wnt-1-like | XP_033728481.1 |
| Mercenaria mercenaria | Wnt-1-like | XP_045178483.1 |
| Haliotis rubra | Wnt-1-like | XP_046558335.1 |
| Bulinus truncatus | Wnt-1 | KAH9487933.1 |
| Octopus sinensis | Wnt-1-like | XP_029650103.2 |
| Euprymna scolopes | Wnt-1 | ABD16194.1 |
| Platynereis dumerilii | Wnt-1 | CAD37164.2 |
| Daphnia magna | wingless | XP_045025225.1 |
| Daphnia pulex | wingless-like | XP_046441682.1 |
| Copidosoma floridanum | Wnt-1 | XP_014212755.1 |
| Cyphomyrmex costatus | Wnt-1 | KYN01512.1 |
| Trichogramma pretiosum | Wnt-1 | XP_014236540.1 |
| Apis florea | Wnt-1 | XP_031776435.1 |
| Crassostrea gigas | Wnt-2b-A | XP_011444401.1 |
| Crassostrea virginica | Wnt-2b-A-like | XP_022296940.1 |
| Ostrea edulis | Wnt-2b-A-like | XP_048739480.1 |
| Mytilus californianus | Wnt-2b-A-like | XP_052057623.1 |
| Pecten maximus | Wnt-2b-A-like | XP_033745012.1 |
| Mytilus edulis | Wnt2 | CAG2218402.1 |
| Mizuhopecten yessoensis | Wnt-2b-A-like | XP_021362612.1 |
| Octopus bimaculoides | Wnt-2b | XP_014786335.1 |
| Lingula anatina | Wnt-2b-A | XP_013384144.1 |
| Octopus sinensis | Wnt-2b-like | XP_029643297.1 |
| Platynereis dumerilii | Wnt-2 | CAD37165.2 |
| Aplysia californica | Wnt-2 | XP_035825355.1 |
| Mytilus coruscus | Wnt-2 | CAC5393656.1 |
| Mercenaria mercenaria | Wnt-2b-A-like | XP_053378281.1 |
| Patella vulgata | Wnt-2b-like | XP_050414918.1 |
| Nanorana parkeri | Wnt-2b | XP_018416347.1 |
| Gallus gallus | Wnt-2b precursor | NP_989667.3 |
| Eudromia elegans | Wnt-2b | NXA42337.1 |
| Pomacea canaliculata | Wnt-2b-like | XP_025107293.1 |
| Haliotis rubra | Wnt-2b-like | XP_046575295.1 |
